# Palliative care case management in a surgical department for patients with gastrointestinal cancer—a register-based cohort study

**DOI:** 10.1007/s00520-024-08794-8

**Published:** 2024-08-16

**Authors:** Stine Gerhardt, Kirstine Skov Benthien, Suzanne Herling, Marie Villumsen, Peter-Martin Krarup

**Affiliations:** 1 Digestive Disease Center, Copenhagen University Hospital – Bispebjerg, Bispebjerg Bakke 23, 2400 Copenhagen, NV Denmark; 2 Palliative Care Unit, Copenhagen University Hospital – Hvidovre, Hvidovre, Denmark; 3 REHPA – Danish Knowledge Centre for Rehabilitation and Palliative Care, Nyborg, Denmark; 4 University of Southern Denmark, Odense, Denmark; 5 The Neuroscience Center, Rigshospitalet, University of Copenhagen, Copenhagen, Denmark; 6 Centre for Clinical Research and Prevention, Copenhagen University Hospital – Frederiksberg, Copenhagen, Denmark

**Keywords:** Palliative care, Case management, Quality of health care, Quality of life, Gastrointestinal neoplasms

## Abstract

**Background:**

The effectiveness of generalist palliative care interventions in hospitals is unknown.

**Aim:**

This study aimed to explore the impact of a palliative care case management intervention for patients with gastrointestinal cancer (PalMaGiC) on hospital admissions, healthcare use, and place of death.

**Design:**

This was a register-based cohort study analyzing data from the Danish Register on Causes of Death, the Danish National Patient Register, and the Danish Palliative Database.

**Setting/participants:**

Deceased patients with gastrointestinal cancer from 2010 to 2020 exposed to PalMaGiC were compared over three periods of time to patients receiving standard care.

**Results:**

A total of 43,969 patients with gastrointestinal cancers were included in the study, of whom 1518 were exposed to PalMaGiC. In the last 30 days of life, exposed patients were significantly more likely to be hospitalized (OR of 1.62 (95% CI 1.26–2.01)), spend more days at the hospital, estimate of 1.21 (95% CI 1.02–1.44), and have a higher number of hospital admissions (RR of 1.13 (95% CI 1.01–1.27)), and were more likely to die at the hospital (OR of 1.94 (95% CI 1.55–2.44)) with an increasing trend over time. No differences were found for hospital healthcare use.

**Conclusion:**

Patients exposed to the PalMaGiC intervention had a greater likelihood of hospitalizations and death at the hospital compared to unexposed patients, despite the opposite intention. Sensitivity analyses show that regional differences may hold some of the explanation for this. Future development of generalist palliative care in hospitals should focus on integrating a home-based approach, community care, and PC physician involvement.

## Background

Gastrointestinal cancers account for 35% of cancer-related deaths worldwide, with a projected 15% increase in mortality within a decade [1]. Patients with end-stage cancers in the esophagus, stomach, pancreas, bile ducts, colon, or rectum often suffer from severe symptoms (1), challenging healthcare systems due to increased healthcare utilization and hospital deaths [2, 3]. Thus, offering effective palliative care (PC) interventions to these patients is crucial.

PC aims to improve the quality of life in patients facing problems related to life-threatening illness and should be delivered in at least two levels: a generalist PC level and a specialized PC (SPC) level [4]. The specialized PC level provides PC for patients with complex needs in specialized PC units or hospices. The generalist PC level delivers PC as an integral approach within the non-specialized setting, such as a surgical hospital department [4, 5]. Achieving a high quality of life at the end of life for patients with life-threatening diseases highly depends on the quality of the PC received [6–8].

Indicators of quality of end-of-life care from administrative data have previously been identified as reduced hospitalizations and healthcare use in the last 30 days of life and decreased mortality in emergency care settings [9, 10]. Additionally, it involves avoiding chemotherapy within the last 14 days of life [10]. Reduced healthcare utilization in PC is associated with improved health-related quality of life (HRQoL), and as Prigerson et al. demonstrated, palliative chemotherapy was associated with reduced HRQoL [11]. Furthermore, patients receiving palliative chemotherapy or emergency surgery had higher rates of hospital admissions and were less likely to die in their preferred location [11].

SPC capacity recommendations in Europe are 80–100 beds per 1 million inhabitants and one specialized PC outpatient team per 100,000 inhabitants [12]. Denmark has 48 beds in SPC per million inhabitants, emphasizing the need for effective PC interventions in hospital departments to provide high-quality PC to all patients not offered SPC [12, 13].

In 2013, a nurse-led palliative care case management intervention for patients with gastrointestinal cancer (PalMaGiC) was implemented in a surgical gastroenterology department (Digestive Disease Center) at the Copenhagen University Hospital – Bispebjerg, Denmark, to improve the quality of generalist PC provided.

To our knowledge, no studies investigated the impact of a similar generalist PC intervention on the quality of end-of-life care [14–17].

Therefore, this study aimed to explore the impact of the PalMaGiC intervention on the quality of end-of-life care, including hospital admissions, healthcare use, and place of death.

## Methods

### Design

This study was a register-based cohort study with retrospective planning, real-time administrative data, and a prospective chronology of exposure and outcomes.

### The cohort

The cohort consisted of all Danish patients above 18 years who died from cancer in the esophagus, cardia, pancreas, stomach, bile duct, liver, ileum, colon, or rectum from 2010 to 2020, identified in the Danish Register on Causes of Death. Cause of death was grouped as (1) esophagus, cardia, and stomach (ECS), (2) pancreas, (3) liver and bile ducts, (4) colon and rectum (colorectal), and (5) ileum or “other digestive cancers” (other). Within this cohort, we defined a group of exposed patients who received the PalMaGiC intervention as patients with any hospital contact with the Digestive Disease Center at the Copenhagen University Hospital – Bispebjerg (DDC) at least 30 days before death, corresponding to the study period of the last 30 days of life. Unexposed were patients in the cohort with any contact with a hospital department outside of the DDC at least 30 days before death who received standard care.

We linked the cohort using the unique personal identification number to The Danish National Patient Register, Statistics Denmark, and the Danish Palliative Database.

### Setting and standard care

The Danish healthcare system is financed through taxes and is free of charge for all patients. In Denmark, everyone with a life-threatening illness should be offered PC based on the needs of the individual [18]. The regions and municipalities are responsible for delivering generalist PC in hospitals, at home, or in nursing homes. In case of complex PC needs, patients can be referred to a specialized PC unit by any medical doctor (GP or specialized). In Denmark, the organization of generalist PC is not subject to politically defined or clinical guidelines.

### The PalMaGiC intervention

All patients at the DDC who were recently diagnosed with incurable gastrointestinal cancer are affiliated with PalMaGiC and offered generalist PC based on their needs. PalMaGiC was initiated in August 2013 to strengthen the quality of generalist PC offered in the DDC. A specialist nurse conducts individualized needs assessments, care plans, and care coordination from the time of an incurable diagnosis of gastrointestinal cancer until death or until initiation of SPC. Patient contacts are by telephone or in the outpatient clinic, depending on patients’ needs. Furthermore, patients have 24-h on-call access to telephone consultation and the possibility of self-referred hospital admission to the DDC if required. Patients can be referred to SPC if they have complex palliative needs.

PalMaGiC was implemented between August 1, 2013, and July 31, 2014. In August 2018, a questionnaire European Organization of Research and Treatment of Cancer QoL Questionnaire Core-15 Palliative Care (EORTC QLQ-C15-PAL) [19] was implemented as a tool to screen and manage symptoms systematically, which was recommended by the Danish Health Authorities [18]. The questionnaire was used as a dialogue tool at the initial contact with PalMaGiC. PalMaGiC serves approximately 120 patients annually and is described in detail in a previous study [20]. The DDC treats approximately 20,000 patients annually.

### Outcomes

In the Danish National Patient Register, we identified the following outcomes that occurred within the last 30 days of life: hospital admissions (yes/no), defined as an overnight stay of at least 8 h; the total number of hospital admissions; length of stay in the hospital, defined as the cumulative number of days between in-patient admission and hospital discharge; the rate of surgery, defined as any surgical procedure (yes/no) except purely palliative interventions such as catheters or stent placements; and radiological examinations, defined as CT, PET CT, and MRI scans (yes/no). Furthermore, antineoplastic treatment, which was chemotherapy or immunotherapy (yes/no) within the last 14 days of life, was retrieved. From the Danish Register on Causes of Death, information about death at the hospital (yes/no) was retrieved. Patients were followed for the last 30 days of life, except for antineoplastic treatment, which was the last 14 days of life.

### Covariates

Covariates included age, gender, education level, cohabiting status, region of residence obtained from Statistics Denmark, comorbidity from the National Patient Register, and, lastly, status on SPC admittance (yes/no) from the Danish Palliative Database. Education level was categorized according to the International Standard Classification of Education system, combining upper secondary and post-secondary: (1) primary and lower secondary school, (2) upper secondary and post-secondary (vocational), (3) short tertiary and bachelor’s level, (4) Master’s level or above, and (5) not classified [21]. Cohabiting status was categorized as (1) widow, (2) divorced/separated, (3) married/living with a partner, and (4) never married. Comorbidity scores were calculated according to the Charlson Comorbidity Index (CCI) based on ICD-10 [22] diagnoses registered from 1998 until death, excluding the cause of death in the calculated score. We grouped CCI scores as < 2 and ≥ 2. The regions of residence were the North Denmark Region, the Central Denmark Region, the Region of Southern Denmark, Region Zealand, and the Capital Region of Denmark.

### Statistical analyses

**Fig. 1:**
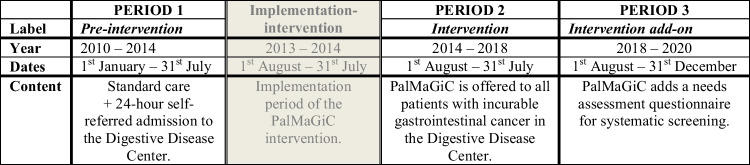
PalMaGiC intervention timeline

To capture trends over time, a timeline with three periods was established: period (1) 2010–2014 (1st of January–31st of July) covering the pre-intervention period, period (2) 2014–2018 (1st of August–31st of July) was defined as the intervention period, and finally period (3) 2018–2020 (1st of August–31st of December) covering the intervention add-on period including the needs-assessment questionnaire. The implementation period (August 1, 2013, and July 31, 2014) was included in the pre-intervention period in the analyses. In the pre-intervention period, exposure consists of cohort patients treated at the Copenhagen University Hospital – Bispebjerg. For details of the timeline, see Fig. 1.

Descriptive statistics were used to present patient characteristics of the study population and outcomes (percentage, median, and range) for each period. Secondly, we created a separate model for each outcome and period to compare the exposed and those unexposed.

The distribution of the variable length of hospital stay was zero-inflated. Therefore, we analyzed the difference in median length of stay and 95% confidence intervals (CI) using the negative binomial distribution. In the adjusted analyses, we included age (continuous), gender, cause of death, CCI, education level, cohabitation status, and admission to SPC.

Crude and adjusted logistic regression analyses were used to investigate the association between the exposure group and the binary outcomes (yes/no): hospital admission, radiological examinations, surgical procedures, and antineoplastic treatment. From the logistic regression analyses, odds ratio (OR) and 95% CI were calculated. Crude and adjusted Poisson regression analyses with log link function were used to investigate the number of hospital admissions as count data. We estimated the relative risk (RR) and corresponding 95% CIs.

Several sensitivity analyses were conducted: all outcomes for the implementation period of 2013–2014 were analyzed to reveal a potentially different trend in the outcomes that would significantly affect the final analyses. Secondly, we tested the interaction between the PalMaGiC intervention periods to determine the difference between the exposed and the unexposed. Finally, we aimed to test whether the differences seen between the DDC and the rest of the country were due to the practice at the DDC alone or whether they resulted from differences caused by the record systems used or regional cultures in how inclined physicians are to admit patients. Therefore, we performed a sensitivity analysis only, including data from the Capital Region of Denmark and the Region of Zealand because the Epic electronic patent record is used in these two regions. The statistical analyses were conducted in SAS 9.4.

## Results

Between 2010 and 2020, in total, 43,969 patients aged 18 or older died from gastrointestinal cancers in the esophagus, cardia, stomach, pancreas, liver, bile ducts, colon, or rectum. Out of the total, 1518 patients were exposed to the PalMaGiC intervention. The characteristics of the exposed and unexposed patients are shown in Table 1.

**Table 1:** Patient characteristics for exposed and unexposed

*N* = 43,969	Exposed *N* = 1518	Unexposed *N* = 42,451
Age, median (range)	75 (25–104)	74 (18–107)
Sex, *n* (%)		
Female	627 (41%)	18,046 (43%)
Cause of death		
Esophagus, cardia, stomach (ECS)	258 (17%)	8170 (19%)
Pancreas	342 (23%)	9973 (23%)
Liver and bile ducts	219 (14%)	5349 (13%)
Colorectal	673 (44%)	18,003 (42%)
Other (Ileum, other digestive)	26 (2%)	872 (2%)
Charlson Comorbidity Index score, *n* (%)		
< 2	877 (58%)	26,346 (62%)
≥ 2	641 (42%)	16,021 (38%)
Education level, *n* (%)		
Primary school lower secondary	509 (34%)	17,140 (40%)
Upper secondary & post-secondary (vocational)	550 (36%)	16,224 (38%)
Short tertiary & and bachelor's level	216 (14%)	5599 (13%)
Master’s level or above	115 (7.5%)	1698 (4%)
Cohabitation status, *n* (%)		
Widowed	351 (23%)	9800 (23%)
Divorced/separated	335 (22%)	5958 (14%)
Married or with a partner	549 (36%)	22,654 (51%)
Not married	266 (18%)	3834 (9%)

Charlson Comorbidity Index scores of < 2 = normal–moderate and ≥ 2 = severe

### Hospitalization in the last 30 days of life and hospital deaths

The proportion of patients hospitalized in the pre-intervention period and throughout the two intervention periods was significantly higher in exposed patients than the unexposed patients (Table 2). There was a trend towards higher hospitalization rates over time in the adjusted analyses, with the intervention add-on period representing the highest difference, with 73% hospitalized in the exposed patients vs. 64% for the unexposed patients (OR of 1.62 (95% CI 1.26–2.01)) (Table 2).

**Table 2:** Hospitalization and hospital deaths

Hospitalization and hospital deaths *Exposed vs. unexposed (ref)*		Pre-intervention		Intervention		Intervention add-on	
		Exposed *N* = 652	Unexposed *N* = 17.248	Exposed *N* = 540	Unexposed *N* = 15.734	Exposed *N* = 326	Unexposed *N* = 9385
Hospitalized within the last 30 days OR (95% CI)	*n* (%)	412 (63%)	10.712 (62%)	350 (65%)	9363 (60%)	238 (73%)	6001 (64%)
	Crude	1.06 (0.90–1.25)		1.25 (1.05–1.50)		1.53 (1.19–1.96)	
	Adjusted***	1.20 (1.02–1.42)		1.35 (1.12–1.62)		1.62 (1.26–2.01)	
Hospital admissions within the last 30 days RR (95% CI)	Median (range)	1 (0–4)	1 (0–7)	1 (0–3)	1 (0–6)	1 (0–4)	1 (0–6)
	Crude	1.00 (0.92–1.09)		1.08 (0.99–1.19)		1.11 (0.99–1.24)	
	Adjusted**	1.06 (0.97–1.16)		1.13 (1.03–1.24)		1.13 (1.01–1.27)	
Length of stay estimate (95% CI)	Median (range)	3 (0–30)	3 (0–30)	4.5 (0–30)	3 (0–30)	5 (0–30)	3 (0–30)
	Crude	1.09 (0.96–1.24)		1.26 (1.10–1.46)		1.20 (1.01–1.42)	
	Adjusted*	1.16 (1.02–1.31)		1.29 (1.12–1.49)		1.21 (1.02–1.44)	
Hospital deaths OR (95% CI)	*n* (%)	335 (51%)	7923 (46%)	288 (53%)	6155 (39%)	160 (49%)	3140 (34%)
	Crude	1.24 (1.06–1.45)		1.78 (1.50–2.11)		1.91 (1.54–2.39)	
	Adjusted***	1.43 (1.21–1.68)		1.92 (1.60–2.29)		1.94 (1.55–2.44)	

Exposed: Patients exposed to PalMaGiC—palliative care case management intention for gastrointestinal cancer. Unexposed: Patients with gastrointestinal cancer are in standard care

*Crude and adjusted negative binomial regression analysis adjusted for age, gender, diagnosis, Charlson Comorbidity Index, education level, cohabitation status, and admission to specialized palliative care

**Crude and Adjusted Poisson regression analysis adjusted for age, gender, diagnoses, Charlson Comorbidity Index, education level, cohabitation status, and admission to specialized palliative care

***Crude and Adjusted logistic regression analysis adjusted for age, gender, diagnoses, Charlson Comorbidity Index, education level, cohabitation status, and admission to specialized palliative care

There was a median length of hospital stay before the intervention of 3 days (0–30) in both the exposed and the unexposed patients. In the adjusted negative binomial regression analysis, however, the exposed patients had significantly longer stays at the hospital, estimate 1.16 (95% CI 1.02–1.31). In the intervention period, the median length of stay was 4.5 (0–30) days for the exposed patients and 3 (0–30) for the unexposed, which was significantly different, estimate of 1.29 (95% CI 1.12–1.49). In the intervention add-on period, the PalMaGiC also had significantly longer median lengths of stay than the unexposed, corresponding to an adjusted estimate of 1.21 (95% CI 1.02–1.44).

Before the intervention, there was no difference between exposed and unexposed for the number of hospital admissions in the last 30 days of life. The PalMaGiC patients had more hospital admissions than the unexposed, with an RR of 1.13 (95% CI 1.03–1.24) in the intervention period. The same applied to the intervention add-on period, where the exposed patients had a RR of 1.13 (95% CI 1.01–1.27) compared to the unexposed.

Dying in the hospital was more frequent in the patients exposed to PalMaGiC in all three time periods, but with an increasing difference between exposed and unexposed patients over time (Table 2). Before the intervention, hospital deaths were 51% vs 46%, corresponding an OR of 1.43 (95% CI 1.21–1.68), in the intervention period 53% vs. 39% with an OR of 1.92 (95% CI 1.60–2.29), and in the intervention add-on period 49% vs. 34% with an OR of 1.94 (95% CI 1.55–2.44).

### Hospital healthcare use in the last 30 days of life

Before the intervention, we observed that 9% of patients exposed to PalMaGiC vs. 12% in the unexposed patients had surgery in the last 30 days of life; this was 9% vs. 10% in the intervention period, and 7% vs. 8% in the intervention add-on period. In the adjusted logistic regression analyses, no differences were observed between exposed and unexposed patients in the pre-intervention period (OR 0.78 (95% CI 0.59–1.04)) or the intervention add-on period (OR 0.87 (95% CI 0.56–1.35)) (Table 3). Similarly, there were no differences in the rate of radiological examinations in the last 30 days of life between exposed and unexposed patients. No differences were observed in antineoplastic treatment in the last 14 days of life, which remained at 2–3% throughout the three periods (Table 3).

**Table 3:** Hospital healthcare use

Hospital healthcare use, *n* (%) *Exposed vs. unexposed *Odds ratio (95% CI)		Pre-intervention		Intervention		Intervention add-on	
		Exposed *N* = 652	Unexposed *N* = 17.248	Exposed *N* = 540	Unexposed *N* = 15.734	Exposed *N* = 326	Unexposed *N* = 9.385
Surgery Last 30 days of life	*n* (%)	56 (9%)	1.993 (12%)	46 (9%)	1.537 (10%)	23 (7%)	727 (8%)
	Crude	0.72 (0.55–0.96)		0.86 (0.63–1.17)		0.90 (0.59–1.39)	
	Adjusted*	0.78 (0.59–1.04)		0.94 (0.69–1.28)		0.87 (0.56–1.35)	
Radiological examinations Last 30 days of life	*n* (%)	248 (38%)	6.554 (38%)	200 (37%)	6.451 (41%)	147 (45%)	3.848 (41%)
	Crude	1.02 (0.87–1.20)		0.87 (0.73–1.04)		1.17 (0.93–1.46)	
	Adjusted*	1.17 (0.99–1.38)		0.94 (0.78–1.13)		1.21 (0.96–1.52)	
Antineoplastic treatment Last 14 days of life	*n* (%)	17 (3%)	581 (3%)	13 (2%)	439 (3%)	10 (3%)	200 (2%)
	Crude	0.77 (0.47–1.26)		0.86 (0.49–1.50)		1.45 (0.76–2.77)	
	Adjusted*	0.90 (0.55–1.49)		1.06 (0.60–1.88)		1.60 (0.83–3.11)	

Exposed: Patients exposed to PalMaGiC—palliative care case management for gastrointestinal cancer. Unexposed: patients with gastrointestinal cancer in standard care

*Crude and Adjusted logistic regression analysis adjusted for age, gender, diagnosis, Charlson Comorbidity Index, education level, cohabitation status, and admission to specialized palliative care

### Sensitivity analyses

The sensitivity analyses of all the outcomes for the PalMaGiC implementation period of 2013–2014 showed no significant differences between the exposed and unexposed. The interaction between the intervention periods revealed a significantly increased likelihood of hospitalizations for exposed patients over time (*p* = 0.04). The sensitivity analysis comparing DDC with the Capital Region of Denmark and the Region of Zealand showed that DDC is not different from these regions when it comes to the number of hospitalizations, and therefore, we must expect that some of the differences we see between the DDC and the rest of the country are explained by regional differences.

## Discussion

This study investigated a generalist PC intervention directed at patients with gastrointestinal cancers in a surgical department. The study found that patients exposed to PalMaGiC were more likely to be hospitalized in the last 30 days of life, with longer stays and a greater chance of dying in the hospital. No significant differences were found in the rate of surgery, radiological examinations, or antineoplastic treatments at the end-of-life between the two groups. To our knowledge, this is the first study to examine the impact of a generalist PC intervention incorporated in a hospital department on the quality of end-of-life outcomes in a large population-based study design.

Previously, RCTs have demonstrated that SPC reduces hospitalization and death in the hospital, but only within the SPC setting [23]. Surprisingly, this study revealed the opposite finding in a generalist PC setting, showing that patients in the exposure group had an increased likelihood of hospitalization over time. Different mechanisms may explain this result.

Before PalMaGiC, patients with incurable cancer affiliated with the DDC could self-refer for hospital admission directly to the department in case of urgent needs. This practice may explain the higher likelihood of hospitalization in the pre-intervention period. PalMaGiC increased the attention to the patient’s needs and symptoms, and symptom screening intensified this focus in the intervention add-on period. However, this inadvertently may have contributed to the increased hospitalizations observed as patients could call the specialist nurse directly during weekdays. Patients consider the PalMaGiC nurse as their main healthcare contact, resulting in more hospital contacts as opposed to primary care contacts without this opportunity [24]. In a previous study focusing on a qualitative evaluation of PalMaGiC, patients highlighted that the essential component of the intervention was easy access to a designated nurse and the personalized approach [24]. The importance of this aspect has been confirmed in other studies [25, 26]. Continuity of care by a few providers is regarded as a quality indicator of end-of-life care [27]. McCaffrey et al. conducted a systematic review to investigate healthcare values essential to the quality of life in patients receiving PC [25]. They found that crucial elements included ready access to care, coordination, continuity, and consistency among healthcare professionals [25].

Another explanation could be that PalMaGiC was delivered through needs-based telephone follow-up and in-hospital meetings. Admitting patients to the department may have been considered the best or most well-known option to alleviate the patient’s symptoms since the possibilities of community nursing vary.

Given the evidence that SPC interventions lead to decreased hospitalization and hospital deaths [23], discussing these opposite findings in the present study is essential. In this cohort, 50% of patients were referred to SPC, assuming that PalMaGiC takes care of the rest of the patients with basic PC needs. In Denmark, SPC is delivered by multidisciplinary teams led by a PC physician in a combination of outpatient clinics, in-hospital, or home-based, depending on patient needs [12]. A systematic review of cohort and quasi-experimental studies investigated the effects of home-based SPC on hospitalization during the last 6 months to 14 days of life [28]. All nineteen studies included in the review, except for one, demonstrated lower hospitalization rates for patients receiving SPC. Despite the high resource consumption, healthcare costs were lower in the intervention groups [28]. We must further explore whether increased collaboration with community nurses and general practitioners can strengthen the PalMaGiC in terms of preventing hospitalizations and deaths at the hospital. However, current differences in community organizations and structures in Denmark may cause significant challenges [29]. Previous studies suggest that community healthcare services may decrease hospitalizations and deaths at the hospital (34, 35). Compared to SPC, where doctors and nurses specialize in PC, PalMaGiC is a nurse-led intervention that collaborates with non-PC physicians specializing in surgery. The PalMaGiC nurse collaborates with physicians on a needs-based approach in surgical and medical treatment decisions. Timely cessation of unnecessary medical treatment and diagnostics is a core component of SPC, which could be difficult for nurses to implement without a physician. This is confirmed in several studies on nurse-led PC interventions, concluding no effects on HRQoL and symptoms of anxiety and depression, possibly due to the lack of physician involvement [16, 17, 30, 31]. When adjusting PalMaGiC, we must consider the impact of the lack of PC-physician support despite the previous studies not being directly comparable to PalMaGiC. Finally, previous research addressed inequity in admittance to SPC [32–34]. Admittance to SPC is lower among men and older patients [33], patients living alone [34], and patients with lower education and income [32], indicating that patients receiving SPC differ from patients receiving generalist PC in terms of age, gender, and socioeconomic status, which might also affect hospitalization rates [35, 36].

Overall, the increased attention to patients’ symptoms and problems following the implementation of PalMaGiC, in combination with a lack of PC-physician involvement, easy access to a designated healthcare contact, and limited community care collaboration, could explain the increased likelihood of hospitalizations and death at the hospital found in this study. It is thus essential to find a balance in meeting the needs of patients for healthcare consistency and easy access to support and care without compromising the quality of PC in terms of an increase in hospitalizations and hospital deaths.

### Strengths and limitations

The register-based design strengthened the study with real-time data using a nationwide population of patients with gastrointestinal cancer. A limitation is the potential unmeasured confounders. It was not possible to fully account for the significance of other healthcare system components, including the role of general practitioners, distance to the hospital, and regional variation in a healthcare organization. The sensitivity analysis demonstrated that the difference between the exposed and unexposed may partly be explained by regional characteristics, which could not be fully accounted for since exposure was confined to one region. This may have overestimated the impact of unwanted exposure. Another limitation is that this study reports on administrative indicators of end-of-life care. Danish registers do not provide data to conclude on the individual patient’s experienced symptom burden, health status, and quality of life.

### Implications for future research and practice

Integrating a home-based approach, PC-physician consultations, and a strengthened collaboration with community nurses and general practitioners should be a focus in developing generalist PC interventions in general hospital departments, such as PalMaGiC. Furthermore, future research should aim to identify patients with gastrointestinal cancers who are at a higher risk of being hospitalized toward the end of life and ultimately dying in the hospital. This may enable PalMaGiC to allocate resources and attention to those patients who require it the most.

## Conclusion

Patients exposed to the PalMaGiC intervention had a greater likelihood of hospitalizations and death at the hospital compared to unexposed patients, which, to some extent, could be attributed to regional differences. Increased attention to patients’ symptoms, easy access to a designated healthcare contact provided by the PalMaGiC, limited community care collaboration, and a lack of PC-physician involvement might also contribute to the results. Future research, adjustments, and development of generalist PC interventions in hospitals may benefit from integrating a home-based approach, community care service, and PC-physician consultations for patients at a high risk of hospitalizations and death at the hospital.

## Data Availability

No datasets were generated or analysed during the current study.

## References

[1] Arnold M, Abnet CC, Neale RE, Vignat J, Giovannucci EL, McGlynn KA et al (2020) Global burden of 5 major types of gastrointestinal cancer. Gastroenterology (New York, NY 1943) 159(1):335–49.e15. 10.1053/j.gastro.2020.02.068 10.1053/j.gastro.2020.02.068 PMC8630546 32247694

[2] Merchant SJ, Brogly SB, Booth CM, Goldie C, Nanji S, Patel SV et al (2019) Palliative care and symptom burden in the last year of life: a population-based study of patients with gastrointestinal cancer. Ann Surg Oncol 26(8):2336–2345. 10.1245/s10434-019-07320-z 30969388 10.1245/s10434-019-07320-z

[3] Okafor PN, Stobaugh DJ, Nnadi AK, Talwalkar JA (2017) Determinants of palliative care utilization among patients hospitalized with metastatic gastrointestinal malignancies. Am J Hosp Palliat Med 34(3):269–274. 10.1177/1049909115624373 10.1177/1049909115624373 26718956

[4] Payne S, Harding A, Williams T, Ling J, Ostgathe C (2022) Revised recommendations on standards and norms for palliative care in Europe from the European Association for Palliative Care (EAPC): A Delphi study. Palliat Med 36(4):680–697. 10.1177/02692163221074547 35114839 10.1177/02692163221074547 PMC9006395

[5] World Health Organization (WHO). Palliative Care 2020 [https://www.who.int/news-room/fact-sheets/detail/palliative-care Palliative Care. (2020). Accessed January 2024.

[6] Beijer S, Kempen GIJM, Pijls-Johannesma MCG, de Graeff A, Dagnelie PC (2008) Determinants of overall quality of life in preterminal cancer patients. Int J Cancer 123(1):232–235. 10.1002/ijc.23497 18412247 10.1002/ijc.23497

[7] Pasman HRW, Brandt HE, Deliens L, Francke AL (2009) Quality indicators for palliative care: a systematic review. J Pain Symptom Manage 38(1):145–56.e21. 10.1016/j.jpainsymman.2008.07.008 19615636 10.1016/j.jpainsymman.2008.07.008

[8] Ostgathe C, Voltz R (2010) Quality indicators in end-of-life care. Curr Opin Support Palliat Care 4(3):170–173. 10.1097/spc.0b013e32833add10 20489644 10.1097/SPC.0b013e32833add10

[9] Earle CC, Landrum MB, Souza JM, Neville BA, Weeks JC, Ayanian JZ (2008) Aggressiveness of cancer care near the end of life: is it a quality-of-care issue? J Clin Oncol 26(23):3860–3866. 10.1200/jco.2007.15.8253 18688053 10.1200/JCO.2007.15.8253 PMC2654813

[10] Earle CC, Park ER, Lai B, Weeks JC, Ayanian JZ, Block S (2003) Identifying potential indicators of the quality of end-of-life cancer care from administrative data. J Clin Oncol 21(6):1133–1138. 10.1200/jco.2003.03.059 12637481 10.1200/JCO.2003.03.059

[11] Prigerson HG, Bao Y, Shah MA, Paulk ME, LeBlanc TW, Schneider BJ et al (2015) Chemotherapy use, performance status, and quality of life at the end of life. JAMA Oncol 1(6):778–784. 10.1001/jamaoncol.2015.2378 26203912 10.1001/jamaoncol.2015.2378 PMC4828728

[12] Timm H, Mikkelsen TB, Jarlbæk L (2017) [Specialized palliative care in Denmark lacks capacity and accessibility]. Ugeskr Laeger. 179(26) 28648170

[13] Rigsrevision: Access to specialist palliative care. (2020). Accessed

[14] Uitdehaag MJRNP, van Putten PGMD, van Eijck CHJMDP, Verschuur EMLRNP, van der Gaast AMDP, Pek CJRNMN et al (2014) Nurse-led follow-up at home vs. conventional medical outpatient clinic follow-up in patients with incurable upper gastrointestinal cancer: A randomized study. J Pain Symptom Manag 47(3):518–30. 10.1016/j.jpainsymman.2013.04.006 10.1016/j.jpainsymman.2013.04.006 23880585

[15] Schenker Y, Althouse AD, Rosenzweig M, White DB, Chu E, Smith KJ et al (2021) Effect of an oncology nurse–led primary palliative care intervention on patients with advanced cancer: the CONNECT cluster randomized clinical trial. JAMA Intern Med 181(11):1451–1460. 10.1001/jamainternmed.2021.5185 34515737 10.1001/jamainternmed.2021.5185 PMC8438619

[16] McCorkle R, Jeon S, Ercolano E, Lazenby M, Reid A, Davies M et al (2015) An advanced practice nurse coordinated multidisciplinary intervention for patients with late-stage cancer: a cluster randomized trial. J Palliat Med 18(11):962–969. 10.1089/jpm.2015.0113 26305992 10.1089/jpm.2015.0113 PMC4638201

[17] Reinke LF, Sullivan DR, Slatore C, Dransfield MT, Ruedebusch S, Smith P et al (2022) A randomized trial of a nurse-led palliative care intervention for patients with newly diagnosed lung cancer. J Palliat Med 25(11):1668–1676. 10.1089/jpm.2022.0008 35649214 10.1089/jpm.2022.0008

[18] Danish Health Authorities: Recommendations for Palliative Care (2017) https://www.sst.dk/da/sygdom-ogbehandling/~/media/79CB83AB4DF74C80837BAAAD55347D0D.ashx2017. Accessed January 2024.

[19] Groenvold M, Petersen MA, Aaronson NK, Arraras JI, Blazeby JM, Bottomley A et al (2006) The development of the EORTC QLQ-C15-PAL: a shortened questionnaire for cancer patients in palliative care. Eur J Cancer 42(1):55–64. 10.1016/j.ejca.2005.06.022 16162404 10.1016/j.ejca.2005.06.022

[20] Gerhardt S, Leerhøy B, Jarlbaek L, Herling S (2023) Qualitative evaluation of a palliative care case management intervention for patients with incurable gastrointestinal cancer (PalMaGiC) in a hospital department. Eur J Oncol Nursing Off J Eur Oncol Nurs Soc 66:102409. 10.1016/j.ejon.2023.102409 10.1016/j.ejon.2023.102409 37742424

[21] UNESCO INSTITUTE FOR STATISTICS: International Standard Classification of Education (ISCED) (2011) https://uis.unesco.org/sites/default/files/documents/international-standard-classification-of-education-isced-2011-en.pdf2011. Accessed 23–01–2024

[22] Thygesen SK, Christiansen CF, Christensen S, Lash TL, Sørensen HT (2011) The predictive value of ICD-10 diagnostic coding used to assess Charlson comorbidity index conditions in the population-based Danish National Registry of Patients. BMC Med Res Methodol 11:83. 10.1186/1471-2288-11-83 21619668 10.1186/1471-2288-11-83 PMC3125388

[23] Kavalieratos D, Corbelli J, Zhang D, Dionne-Odom JN, Ernecoff NC, Hanmer J et al (2016) Association between palliative care and patient and caregiver outcomes: a systematic review and meta-analysis. JAMA, J Am Med Assoc 316(20):2104–2114. 10.1001/jama.2016.16840 10.1001/jama.2016.16840 PMC5226373 27893131

[24] Gerhardt S, Leerhøy B, Jarlbaek L, Herling S (2023) Qualitative evaluation of a palliative care case management intervention for patients with incurable gastrointestinal cancer (PalMaGiC) in a hospital department. Eur J Oncol Nurs 66:102409. 10.1016/j.ejon.2023.102409 37742424 10.1016/j.ejon.2023.102409

[25] McCaffrey N, Bradley S, Ratcliffe J, Currow DC (2016) What aspects of quality of life are important from palliative care patients’ perspectives? A systematic review of qualitative research. J Pain Symptom Manage 52(2):318–28.e5. 10.1016/j.jpainsymman.2016.02.012 27216362 10.1016/j.jpainsymman.2016.02.012

[26] Virdun C, Luckett T, Lorenz K, Davidson PM, Phillips J (2020) Hospital patients’ perspectives on what is essential to enable optimal palliative care: a qualitative study. Palliat Med 34(10):1402–1415. 10.1177/0269216320947570 32857012 10.1177/0269216320947570

[27] Grunfeld E, Urquhart R, Mykhalovskiy E, Folkes A, Johnston G, Burge FI et al (2008) Toward population-based indicators of quality end-of-life care: testing stakeholder agreement. Cancer 112(10):2301–2308. 10.1002/cncr.23428 18361447 10.1002/cncr.23428 PMC3749155

[28] Gonzalez-Jaramillo V, Fuhrer V, Gonzalez-Jaramillo N, Kopp-Heim D, Eychmüller S, Maessen M (2021) Impact of home-based palliative care on health care costs and hospital use: a systematic review. Palliat Support Care 19(4):474–487. 10.1017/S1478951520001315 33295269 10.1017/S1478951520001315

[29] Knowledge Center for Rehabilitation and Palliative Care in Denmark. Cancer Rehabilitation in Denmark - status and development from 2017–2021. https://www.rehpa.dk/wp-content/uploads/2022/01/Kraeftrehabilitering-FINAL.pdf2022 (2022). Accessed January 2024

[30] Stefan MS, Knee AB, Ready A, Rastegar V, Burgher Seaman J, Gunn B, et al (2022) Efficacy of models of palliative care delivered beyond the traditional physician-led, subspecialty consultation service model: a systematic review and meta-analysis. BMJ supportive & palliative care. bmjspcare-2021–003507 10.1136/bmjspcare-2021-003507 10.1136/bmjspcare-2021-003507 35440488

[31] Schenker Y, Althouse AD, Rosenzweig M, White DB, Chu E, Smith KJ et al (2021) Effect of an oncology nurse-led primary palliative care intervention on patients with advanced cancer: the CONNECT cluster randomized clinical trial. JAMA Intern Med 181(11):1451–1460. 10.1001/jamainternmed.2021.5185 34515737 10.1001/jamainternmed.2021.5185 PMC8438619

[32] Adsersen M, Thygesen LC, Neergaard MA, Sjøgren P, Mondrup L, Nissen JS et al (2023) Higher admittance to specialized palliative care for patients with high education and income: a nationwide register-based study. J Palliat Med 26(1):57–66. 10.1089/jpm.2022.0087 36130182 10.1089/jpm.2022.0087

[33] Adsersen M, Thygesen LC, Jensen AB, Neergaard MA, Sjøgren P, Groenvold M (2017) Is admittance to specialised palliative care among cancer patients related to sex, age and cancer diagnosis? A nation-wide study from the Danish Palliative Care Database (DPD). BMC Palliative Care 16(1):21. 10.1186/s12904-017-0194-z 28330507 10.1186/s12904-017-0194-z PMC5363002

[34] Adsersen M, Thygesen LC, Neergaard MA, Jensen AB, Sjøgren P, Damkier A et al (2019) Cohabitation status influenced admittance to specialized palliative care for cancer patients: a nationwide study from the Danish palliative care database. J Palliat Med 22(2):164–172. 10.1089/jpm.2018.0201 30403554 10.1089/jpm.2018.0201

[35] Chang CM, Wu CC, Yin WY, Juang SY, Yu CH, Lee CC (2014) Low socioeconomic status is associated with more aggressive end-of-life care for working-age terminal cancer patients. The oncologist (Dayton, Ohio) 19(12):1241–1248. 10.1634/theoncologist.2014-0152 10.1634/theoncologist.2014-0152 PMC4257740 25342317

[36] Yu CW, Alavinia SM, Alter DA (2020) Impact of socioeconomic status on end-of-life costs: a systematic review and meta-analysis. BMC Palliative care 19(1):35. 10.1186/s12904-020-0538-y 32293403 10.1186/s12904-020-0538-y PMC7087362

